# Hidden Markov Models and their Applications in Biological Sequence Analysis

**DOI:** 10.2174/138920209789177575

**Published:** 2009-09

**Authors:** Byung-Jun Yoon

**Affiliations:** Department of Electrical & Computer Engineering, Texas A&M University, College Station, TX 77843-3128, USA

**Keywords:** Hidden Markov model (HMM), pair-HMM, profile-HMM, context-sensitive HMM (csHMM), profile-csHMM, sequence analysis.

## Abstract

Hidden Markov models (HMMs) have been extensively used in biological sequence analysis. In this paper, we give a tutorial review of HMMs and their applications in a variety of problems in molecular biology. We especially focus on three types of HMMs: the profile-HMMs, pair-HMMs, and context-sensitive HMMs. We show how these HMMs can be used to solve various sequence analysis problems, such as pairwise and multiple sequence alignments, gene annotation, classification, similarity search, and many others.

## 1. INTRODUCTION

The successful completion of many genome sequencing projects has left us with an enormous amount of sequence data. The sequenced genomes contain a wealth of invaluable information that can help us better understand the underlying mechanisms of various biological functions in cells. However, considering the huge size of the available data, it is virtually impossible to analyze them without the help of computational methods. In order to extract meaningful information from the data, we need computational techniques that can efficiently analyze the data according to sound mathematical principles. Given the expanding list of newly sequenced genomes and the increasing demand for genome re-sequencing in various comparative genomics projects, the importance of computational tools in biological sequence analysis is expected to grow only further.

Until now, various signal processing models and algorithms have been used in biological sequence analysis, among which the hidden Markov models (HMMs) have been especially popular. HMMs are well-known for their effectiveness in modeling the correlations between adjacent symbols, domains, or events, and they have been extensively used in various fields, especially in speech recognition [1] and digital communication. Considering the remarkable success of HMMs in engineering, it is no surprise that a wide range of problems in biological sequence analysis have also benefited from them. For example, HMMs and their variants have been used in gene prediction [2], pairwise and multiple sequence alignment [3, 4], base-calling [5], modeling DNA sequencing errors [6], protein secondary structure prediction [7], ncRNA identification [8], RNA structural alignment [9], acceleration of RNA folding and alignment [10], fast noncoding RNA annotation [11], and many others.

In this paper, we give a tutorial review of HMMs and their applications in biological sequence analysis. The organization of the paper is as follows. In Sec. 2, we begin with a brief review of HMMs and the basic problems that must be addressed to use HMMs in practical applications. Algorithms for solving these problems are also introduced. After reviewing the basic concept of HMMs, we introduce three types of HMM variants, namely, profile-HMMs, pair-HMMs, and context-sensitive HMMs, that have been useful in various sequence analysis problems. Section 3 provides an overview of profile hidden Markov models and their applications. We also introduce publicly available profile-HMM software packages and libraries of pre-built profile-HMMs for known sequence families. In Sec. 4, we focus on pair-HMMs and their applications in pairwise alignment, multiple sequence alignment, and gene prediction. Section 5 reviews context-sensitive HMMs (csHMMs) and profile context-sensitive HMMs (profile-csHMMs), which are especially useful for representing RNA families. We show how these models and other types of HMMs can be employed in RNA sequence analysis.

## 2. HIDDEN MARKOV MODELS

A *hidden Markov model (HMM)* is a statistical model that can be used to describe the evolution of observable events that depend on internal factors, which are not directly observable. We call the observed event a `symbol' and the invisible factor underlying the observation a `state'. An HMM consists of two stochastic processes, namely, an invisible process of hidden states and a visible process of observable symbols. The hidden states form a *Markov chain*, and the probability distribution of the observed symbol depends on the underlying state. For this reason, an HMM is also called a doubly-embedded stochastic process [1].

Modeling observations in these two layers, one visible and the other invisible, is very useful, since many real world problems deal with classifying raw observations into a number of categories, or class labels, that are more meaningful to us. For example, let us consider the speech recognition problem, for which HMMs have been extensively used for several decades [1]. In speech recognition, we are interested in predicting the uttered word from a recorded speech signal. For this purpose, the speech recognizer tries to find the sequence of phonemes (states) that gave rise to the actual uttered sound (observations). Since there can be a large variation in the actual pronunciation, the original phonemes (and ultimately, the uttered word) cannot be directly observed, and need to be predicted.

This approach is also useful in modeling biological sequences, such as proteins and DNA sequences. Typically, a biological sequence consists of smaller substructures with different functions, and different functional regions often display distinct statistical properties. For example, it is well-known that proteins generally consist of multiple domains. Given a new protein, it would be interesting to predict the constituting domains (corresponding to one or more states in an HMM) and their locations in the amino acid sequence (observations). Furthermore, we may also want to find the protein family to which this new protein sequence belongs. In fact, HMMs have been shown to be very effective in representing biological sequences [3], as they have been successfully used for modeling speech signals. As a result, HMMs have become increasingly popular in computational molecular biology, and many state-of-the-art sequence analysis algorithms have been built on HMMs.

### 2.1. Definition

Let us now formally define an HMM. We denote the observed symbol sequence as **x** = *x*_1_*x*_2_ ... *x*_*L*_ and the underlying state sequence as **y** = *y*_1_*y*_2_ ... *y*_*L*_, where *y_n_*. is the underlying state of the ***n*** th observation *x_n_*. Each symbol *x_n_* takes on a finite number of possible values from the set of observations **O** =  {*O*_1_*O*_2_,...,*O*_N_} and each state *y_n_* takes one of the values from the set of states **S** = {1,2,...,*M*}, where *N* and *M* denote the number of distinct observations and the number of distinct states in the model, respectively. We assume that the hidden state sequence is a time-homogeneous first-order Markov chain. This implies that the probability of entering state *j *in the next time point depends only on the current state *i*, and that this probability does not change over time. Therefore, we have

(1) $$P\left\{y_{n+1}=j|y_{n}=i,y_{n-1}=i_{n-1},...,y_{1}=i_{1}\right\}=P\left\{y_{n+1}=j|y_{n}=i\right\}=t\left(i,j\right)$$

for all states *i*, *j* ∈ *S* and for all *n* ≥ 1. The fixed probability for making a transition from state *i* to state *j* is called the *transition probability*, and we denote it by *t*(*i*, *j*). For the initial state *y*_1_, we denote the *initial state probability* as π(*i*)= ***P*** {*y*_1_ = *i*} for all *i* ∈ *S*. The probability that the *n* th observation will be *x*_n_ = *x* depends only on the underlying state *y_n_*, hence

(2) $$P\left\{x_{n}=x|y_{n}=i,y_{n-1},x_{n-1},...\right\}=P\left\{x_{n}=x|y_{n}=i\right\}=e\left(x|i\right)$$

for all possible observations *x* ∈ ***O***, all state *i* ∈ *S*, and all *n* ≥ 1. This is called the *emission probability* of *x* at state *i*, and we denote it by *e*(*x* | *i*). The three probability measures *t*(*i*, *j*), *π*(*i*), and *e*(*x* | *i*) completely specify an HMM. For convenience, we denote the set of these parameters as **Θ**.

Based on these parameters, we can now compute the probability that the HMM will generate the observation sequence **x** = *x*_1_ *x*_2_ ... *x_L_* with the underlying state sequence **y** = *y*_1_ *y*_2_ ... *y_L_*. This joint probability *P* {**x,y | Θ**} can be computed by

(3) $$P\left\{x,y\;|\text{Θ}\right\}=P\left\{x|y,\text{Θ}\right\}P\left\{y|\text{Θ}\right\},$$

where

(4) $$P\left\{x|y,\text{Θ}\right\}=e\left(x_{1}|y_{1}\right)e\left(x_{2}|y_{2}\right)e\left(x_{3}|y_{3}\right)...e\left(x_{L}|y_{L}\right)$$

(5) $$P\left\{y|\text{Θ}\right\}=\pi \left(y_{1}\right)t\left(y_{1},y_{2}\right)t\left(y_{2},y_{3}\right)...t\left(y_{L-1},y_{L}\right).$$

As we can see, computing the observation probability is straightforward when we know the underlying state sequence.

### 2.2. A Simple HMM forModeling Eukaryotic Genes

As we mentioned earlier, HMMs can be effectively used for representing biological sequences. As a simple example, let us consider an HMM that models protein-coding genes in eukaryotes. It is well known that many protein-coding regions display codon bias. The nonuniform usage of codons results in different symbol statistics for different codon positions [12], and it is also a source of the period-3 property in the coding regions [13]. These properties are not observed in introns, which are not translated into amino acids. Therefore, it is important to incorporate these codon statistics when modeling protein-coding genes and building a gene-finder. Fig. (**1**) shows a toy HMM for modeling eukaryotic genes. The given HMM tries to capture the statistical differences in exons and introns. The HMM has four states, where *E*_1_, *E*_2_, and *E*_3_ are used to model the base statistics in exons. Each *E*_k_ uses a different set of emission probabilities to reflect the symbol statistics at the *k* th position of a codon. The state *I* is used to model the base statistics in introns. Note that this HMM can represent genes with multiple exons, where the respective exons can have variable number of codons, and the introns can also have variable lengths. This example shows that if we know the structure and the important characteristics of the biological sequences of interest, building the corresponding HMM is relatively simple and it can be done in an intuitive manner.

The constructed HMM can now be used to analyze new observation sequences. For example, let us assume that we have a new DNA sequence **x** = *x*_1_ ... *x*_19_ = ATGCGACTGCATAGCACTT How can we find out whether this DNA sequence is a coding gene or not? Or, if we assume that **x** is a protein-coding gene, how can we predict the locations of the exons and introns in the given sequence? We can answer the first question by computing the observation probability of **x** based on the given HMM that models coding genes. If this probability is high, it implies that this DNA sequence is likely to be a coding gene. Otherwise, we may conclude that **x** is unlikely to be a coding gene, since it does not contain the statistical properties that are typically observed in protein-coding genes. The second question is about predicting the internal structure of the sequence, as it cannot be directly observed. To answer this question, we may first predict the state sequence **y** in the HMM that best describes **x**. Once we have inferred the best **y**, it is straightforward to predict the locations of the exons and introns. For example, assume that the optimal state sequence **y** is as shown in Fig. (**1**). This implies that the first nine bases *x*_1_ ... *x*_9_ belong to the first exon, the following four bases *x*_10_ ... *x*_13_ belong to an intron, and the last six bases *x*_14_ ... *x*_19_ belong to another exon. As these examples show, HMMs provide a formal probabilistic framework for analyzing biological sequences.

### 2.3. Basic Problems and Algorithms for HMMs

There are three basic problems that have to be addressed in order to use HMMs in practical applications. Suppose we have a new symbol sequence **x** = *x*_1_*x*_2_ ...*x_L_*. How can we compute the observation probability ***P*{x | Θ**} based on a given HMM? This problem is sometimes called the *scoring problem*, since computing the probability *P*{**x | Θ}** is a natural way of `scoring' a new observation sequence **x** based on the model at hand. Note that for a given **x**, its underlying state sequence is not directly observable and there can be many state sequences that yield **x**. Therefore, one way to compute the observation probability is to consider all possible state sequences **y** for the given **x** and sum up the probabilities as follows

(6) $$P\left\{x|\Theta \right\}=\sum_{y}P\left\{\text{x},\text{y}|\Theta \right\}.$$

However, this is computationally very expensive, since there are *M^L^* possible state sequences. For this reason, we definitely need a more efficient method for computing *P*{**x | Θ**}. There exist a dynamic programming algorithm, called the *forward algorithm*, that can compute *P*{**x | Θ**} in an efficient manner [1]. Instead of enumerating all possible state sequences, this algorithm defines the following *forward variable*

(7) $$\alpha \left(n,i\right)=P\left\{x_{1}...x_{n},y_{n}=i|\Theta \right\}.$$

This variable can be recursively computed using the following formula

(8) $$\alpha \left(n,i\right)=\sum_{k}\left[\alpha \left(n-1,k\right)t\left(k,i\right)e\left(x_{n}|i\right)\right],$$

for *n* = 2,...,*L*. At the end of the recursions, we can compute $P\left\{x|\Theta \right\}=\sum_{k}\alpha \left(L,k\right)$ . This algorithm computes the observation probability of **x** with only *O*(*LM*^2^) computations. Therefore, the amount of time required for computing the probability increases only linearly with the sequence length *L* , instead of increasing exponentially.

Another practically important problem is to find the optimal state sequence, or the optimal path, in the HMM that maximizes the observation probability of the given symbol sequence **x**, Among all possible state sequences **y**, we want to find the state sequence that best explains the observed symbol sequence. This can be viewed as finding the best alignment between the symbol sequence and the HMM, hence it is sometimes called the *optimal alignment* problem. Formally, we want to find the optimal path y^*^ that satisfies the following

(9) $$y^{*}=a\underset{y}{\mathrm{rgmax}}P\left\{y|x,\Theta \right\}$$

Note that this is identical to finding the state sequence that maximizes *P*{**x,y | Θ**} , since we have

(10) $$P\left\{y|x,\Theta \right\}=\frac{P\left\{x,y|\Theta \right\}}{P\left\{x|\Theta \right\}}.$$

Finding the optimal state sequence y^*^ by comparing all *M^L^* possible state sequences is computationally infeasible. However, we can use another dynamic programming algorithm, well-known as the *Viterbi algorithm*, to find the optimal path y^*^ efficiently [14, 15]. The Viterbi algorithm defines the variable

(11) $$\gamma \left(n,i\right)=\underset{y_{1},...,y_{n-1}}{\text{max}}P\left\{x_{1}\cdot \cdot \cdot x_{n},y_{1}\cdot \cdot \cdot y_{n-1}y_{n}=i|\Theta \right\},$$

and computes it recursively using the following formula

(12) $$\gamma \left(n,i\right)=\max_{k}\left[\gamma \left(n-1,k\right)t\left(k,i\right)e\left(x_{n}|i\right)\right].$$

At the end, we can obtain the maximum observation probability as follows

(13) $$P^{*}=\underset{y}{\text{max}}P\left\{x,y|\Theta \right\}=\max_{\text{k}}\gamma \left(L,k\right).$$

The optimal path y^*^ can be easily found by tracing back the recursions that led to the maximum probability $P^{*}=P\left\{x,y^{*}|\Theta \right\}$ . Like the forward algorithm, the Viterbi algorithm finds the optimal state sequence in *O*(*LM*^2^) time.

As we have seen, the Viterbi algorithm finds the optimal path that maximizes the observation probability of the *entire* symbol sequence. In some cases, it may be more useful to find the optimal states individually for each symbol position. In this case, we can find the optimal state *y_n_* that is most likely to be the underlying state of *x_n_* as follows

(14) $${\hat{y}}_{n}=a\underset{i}{\mathrm{rgmax}}P\left\{y_{n}=i|x,\Theta \right\},$$

based on the given **x** and Θ. The posterior probability *P*{*y_n_* = *i* | **x, Θ**} can be computed from

(15) $$P\left\{y_{n}=i|x,\Theta \right\}=\frac{P\left\{x_{1}...x_{n},y_{n}=i|\Theta \right\}P\left\{x_{n+1}...x_{L}|y_{n}=i,\Theta \right\}}{P\left\{x|\Theta \right\}}=\frac{\alpha \left(n,i\right)\beta \left(n,i\right)}{\sum_{k}\alpha \left(n,k\right)\beta \left(n,k\right)},$$

where *β*(*n,i*)is defined as

(16) $$\beta \left(n,i\right)=P\left\{x_{n+1}...x_{L}|y_{n}=i,\Theta \right\}.$$

This *backward variable* *β*(*n,i*) can be recursively computed using the *backward algorithm* as follows

(17) $$\beta \left(n,i\right)=\sum_{k}\left[t\left(i,k\right)e\left(x_{n+1}|k\right)\beta \left(n+1,k\right)\right],$$

for *n* = *L* – 1, *L* – 2,…,1. The advantage of predicting the optimal states individually is that this approach will maximize the expected number of correctly predicted states. However, the overall state sequence $\hat{y}=\hat{y_{1}}\hat{y_{2}}...\hat{y_{L}}$ will be generally suboptimal, hence $P\left\{x,\hat{y}|\Theta \right\}\leq P\left\{x,y^{*}|\Theta \right\}$ . In some cases, the predicted path $\hat{y}$ may not be even a legitimate path in the given HMM, in which case we will have $P\left\{x,\hat{y}|\Theta \right\}=0$ . For this reason, the Viterbi algorithm is often preferred when we are interested in inferring the optimal state sequence for the entire observation **x**, while the posterior-decoding approach in (14) is preferred when our interest is mainly in predicting the optimal state at a specific position. The posterior probability in (15) can also be useful for estimating the reliability of a state prediction. For example, we may first predict the optimal path $y^{*}=y_{1}^{*}...y_{L}^{*}$ as in (9) using the Viterbi algorithm, and then estimate the reliability of the individual state prediction $y_{n}^{*}$ by computing the posterior probability $P\left\{y_{n}=y_{n}^{*}|x,\Theta \right\}$ as in (15).

The scoring problem and the alignment problem are concerned about analyzing a new observation sequence **x** based on the given HMM. However, the solutions to these problems are meaningful only if the HMM can properly represent the sequences of our interest. Let us assume that we have a set of related observation sequences **X** = {**x**_1_,**x**_2_,...,**x**_*T*_} that we want to represent by an HMM. For example, they may be different speech recordings of the same word or protein sequences that belong to the same functional family. Now, the important question is how we can reasonably choose the HMM parameters based on these observations. This is typically called the *training problem*. Although there is no optimal way of estimating the parameters from a limited number of finite observation sequences, there are ways to find the HMM parameters that locally maximize the observation probability [1, 16-18]. For example, we can use the *Baum-Welch* algorithm [16] to train the HMM. The Baum-Welch algorithm is an expectation-maximization (EM) algorithm that iteratively estimates and updates Θ based on the *forward-backward* procedure [1, 16]. Since the estimation of the HMM parameters is essentially an optimization problem, we can also use standard gradient-based techniques to find the optimal parameters of the HMM [17, 18]. It has been demonstrated that the gradient-based method can yield good estimation results that are comparable to those of the popular EM-based method [18]. When the precise evaluation of the probability (or likelihood) of an observation is practically intractable for the HMM at hand, we may use simulation-based techniques to evaluate it approximately [17, 19]. These techniques allow us to handle a much broader class of HMMs. In such cases, we can train the HMM using the *Monte Carlo EM (MCEM)* algorithm, which adopts the Monte Carlo approach to approximate the so-called E-step (expectation step) in the EM algorithm [19]. There are also training methods based on stochastic optimization algorithms, such as simulated annealing, that try to improve the optimization results by avoiding local maxima [20, 21]. Currently, there exists a vast literature on estimating the parameters of hidden Markov models, and the reader is referred to [1, 17, 19, 22, 23] for further discussions.

### 2.4. Variants of HMMs

There exist a large number of HMM variants that modify and extend the basic model to meet the needs of various applications. For example, we can add silent states (i.e., states that do not emit any symbol) to the model in order to represent the absence of certain symbols that are expected to be present at specific locations [24, 25]. We can also make the states emit two aligned symbols, instead of a single symbol, so that the resulting HMM simultaneously generates two related symbol sequences [3, 4, 26]. It is also possible to make the probabilities at certain states dependent on part of the previous emissions [9, 27] so that we can describe more complex symbol correlations. In the following sections, we review a number of HMM variants that have been used in various biological sequence analysis problems.

## 3. PROFILE HIDDEN MARKOV MODELS

Let us assume that we have a multiple sequence alignment of proteins or DNA sequences that belong to the same functional family. How can we build an HMM that can effectively represent the common patterns, motifs, and other statistical properties in the given alignment? One model that is especially useful for representing the profile of a multiple sequence alignment is the *profile hidden Markov model (profile-HMM)* [24, 25]. Profile-HMMs are HMMs with a specific architecture that is suitable for modeling sequence profiles. Unlike general HMMs, profile-HMMs have a strictly linear left-to-right structure that does not contain any cycles. A profile-HMM repetitively uses three types of hidden states, namely, *match states M_k_*, *insert states I_k_*, and *delete states D_k_*, to describe position-specific symbol frequencies, symbol insertions, and symbol deletions, respectively.

### 3.1. Constructing a Profile-HMM

To see how profile-HMMs work, let us consider the following example. Suppose we want to construct a profile-HMM based on the multiple alignment shown in Fig. (**2a**).

As we can see, the given alignment has five columns, where the base frequencies in the respective columns are different from each other. The *k* th match state *M_k_* in the profile-HMM is used to describe the symbol frequencies in the *k* th column of the alignment. It is called a `match' state, since it is used to represent the case when a symbol in a new observation sequence matches the *k* th symbol in the consensus sequence of the original alignment. As a result, the number of match states in the resulting profile-HMM is identical to the length of the consensus sequence. The emission probability *e*(*x*|*M_k_*) at the *k* th match state *M_k_* reflects the observed symbol frequencies in the *k* th consensus column. By interconnecting the match states *M*_1_,*M*_2_,...,*M*_5_, we obtain an *ungapped HMM* as shown in Fig. (**2b**). This ungapped HMM can represent DNA sequences that match the consensus sequence of the alignment without any gap, and it serves as the backbone of the final profile-HMM that is to be constructed.

Once we have constructed the ungapped HMM, we add insert states *I_k_* and delete states *D_k_* to the model so that we can account for insertions and deletions in new observation sequences. Let us first consider the case when the observed DNA sequence is longer than the consensus sequence of the original alignment. In this case, if we align these sequences, there will be one or more bases in the observed DNA sequence that are not present in the consensus sequence. These additional symbols are modeled by the insert states. The insert state *I_k_* is used to handle the symbols that are inserted between the *k* th and the (*k* + 1)th positions in the consensus sequence. Now, let us consider the case when the new observed sequence is shorter than the consensus sequence. In this case, there will be one or more bases in the consensus sequence that are not present in the observed DNA sequence. The *k* th delete state *D_k_* is used to handle the deletion of the *k* th symbol in the original consensus sequence. As delete states represent symbols that are missing, *D_k_* is a *non-emitting state*, or a *silent* state, which is simply used as a place-holder that interconnects the neighboring states. After adding the insert states and the delete states to the ungapped HMM in Fig. (**2b**), we obtain the final profile-HMM that is shown in Fig. (**2c**).

Estimating the parameters of a profile-HMM based on a given multiple sequence alignment is relatively simple. We first have to decide which columns should be represented by match states and which columns should be modeled by insert states. Suppose we have a column that contains one or more gaps. Should we regard the symbols in the column as `insertions', or should we rather view the gaps in the column as `deletions'? One simple rule would be to compare the number of symbols and the number of gaps. If the column has more symbols than gaps, we treat the gaps as symbol deletions. Therefore, we model the column using a match state *M_k_* (for the symbols in the given column) and a delete state *D_k_* (for the gaps in the same column). On the contrary, if we have more gaps than symbols, it would make more sense to view the symbols as insertions, hence we use an insert state *I_k_* to represent the column. Once we have decided which columns should be represented by match states and which ones should be represented by insert states, we know the underlying state sequence for each symbol sequence in the alignment. Therefore, we can estimate the transition probabilities and the emission probabilities of the profile-HMM by counting the number of each state transition or symbol emission and computing their relative frequencies. To allow small probability for state transitions or symbol emissions that are not observed in the original alignment, we can add the so-called *pseudocounts* to the actual counts [3].

We can also use more sophisticated methods for parameterizing the profile-HMMs. In fact, there have been considerable research efforts for optimal construction and parameterization of profile-HMMs to improve their overall performance. More discussions on this topic can be found in [3, 28-32].

### 3.2. Applications of Profile-HMMs

Due to the convenience and effectiveness in representing sequence profiles, profile-HMMs have been widely used for modeling and analyzing biological sequences. When profile-HMMs were first proposed, they were quickly adopted for modeling the characteristics of a number of protein families, such as globins, immunoglobulins, and kinases [33]. They have been shown to be useful for various tasks, including protein classification, motif detection, and finding multiple sequence alignments. Nowadays, there exist publicly available software packages, such as HMMER [3] and SAM [34, 35], that can be readily used to build and train profile-HMMs. These packages provide convenient tools for applying profile-HMMs to various sequence analysis problem. A comparison between these two popular HMM packages and an assessment of their critical features can be found in [32].

It would be also very convenient to have a library of ready-made profile-HMMs for known sequence families. Currently, we have two such libraries that have compiled a large number of profile-HMMs for various protein families: the PROSITE database [36, 37] and the Pfam database [38, 39]. Given a profile-HMM that represents a biological sequence family, we can use it to search a sequence database to find additional homologues that belong to the same family. In a similar manner, if we have a database of pre-built profile-HMMs, we can use a single query sequence to search through the database to look for matching profiles. This strategy can be used for classification and annotation of the given sequence. For example, by querying a new protein sequence against Pfam or PROSITE, we can find out whether the sequence contains any of the known protein domains.

Sometimes, we may want to compare two multiple sequence alignments or sequence profiles, instead of comparing a single sequence against a multiple alignment or a profile. Comparing sequence profiles can be beneficial for detecting remote homologues, and profile-HMMs have also been used for this purpose [40-42]. For example, COACH [40] allows us to compare sequence alignments, by building a profile-HMM from one alignment and aligning the other alignment to the constructed profile-HMM. HHsearch [42] generalizes the traditional pairwise sequence alignment algorithm for finding the alignment of two profile-HMMs. Another program, called PRC (profile comparer) [41], provides a tool for scoring and aligning profile-HMMs produced by popular software tools, including HMMER [3] and SAM [34, 35].

Although profile-HMMs have been widely used for representing sequence profiles, their application is by no means limited to modeling amino acid or nucleotide sequences. For example, Di Francesco *et al*. [43, 44] used profile-HMMs to model sequences of protein secondary structure symbols: helix (H), strand (E), and coil (C). Therefore, the model emits only three types of symbols instead of twenty different amino acids. It has been demonstrated that this profile-HMM can be used for recognizing the three-dimensional fold of new protein sequences based on their secondary structure predictions. Another interesting example is the *feature-based profile-HMM* that was proposed to improve the performance of remote protein homology detection [45]. Instead of emitting amino acids, emissions of these HMMs are based on `features' that capture the biochemical properties of the protein family of interest. These features are extracted by performing a spectral analysis of a number of selected `amino acid indices' [46] and using principal component analysis (PCA) to reduce the redundancy in the resulting signal.

There are also variants of the basic profile-HMM, where the *jumping profile-HMM (jpHMM)* [47] is one such example. The jumping profile-HMM is a probabilistic generalization of the so-called *jumping-alignment* approach. The jumping-alignment approach is a strategy for comparing a sequence with a multiple alignment, where the sequence is not aligned to the alignment as a whole, but it can `jump' between the sequences that constitute the alignment. In this way, different parts of the sequence can be aligned to different sequences in the given alignment. A jpHMM uses multiple match states for each column to represent different sequence subtypes. The HMM is allowed to jump between these match states based on the local similarity of the sequence and the different sequence subtypes in the model. This approach has been shown to be especially useful for detecting recombination breakpoints [47].

## 4. PAIR HIDDEN MARKOV MODELS

In biological sequence analysis, it is often important to compare two sequences to find out whether these sequences are functionally related. Sequence similarity is often a good indicator of their functional relevance, and for this reason, methods for quantitatively measuring the similarity of two proteins or DNA sequences have been of interest to many researchers. A typical approach for comparing two biological sequences is to align them based on their similarity, compute their alignment score, and evaluate the statistical significance of the predicted alignment. To find the best alignment between the sequences, we first have to define a reasonable scoring scheme for ranking different alignments. Based on this scoring scheme, we can choose the alignment that maximizes the alignment score.

### 4.1. Pair-HMMs forModeling Aligned Sequence Pairs

The *pair hidden Markov model (pair-HMM)* [3] is a variant of the basic HMM that is especially useful for finding sequence alignments and evaluating the significance of the aligned symbols. Unlike the original HMM, which generates only a single sequence, a pair-HMM generates an aligned pair of sequences. For example, let us consider the pair-HMM shown in Fig. (**3**).

This simple pair-HMM traverses between the states *I_x_*, *I_z_*, and *A* , to simultaneously generate two aligned DNA sequences $x=x_{1}...x_{L_{x}}$ (sequence 1) and $z=z_{1}...z_{L_{z}}$ (sequence 2). The state *I_x_* emits a single unaligned symbol *x_i_* in the first sequence **x**. Similarly, the state *I_z_* emits an unaligned symbol *z_j_* only in the second sequence **z**. Finally, the state *A* generates an aligned pair of two symbols *x_i_* and *z_j_*, where *x_i_* is inserted in **x** and *z_j_* is inserted in **z**. For example, let us consider the alignment between **x** = *x*_1_ *x*_2_*x*_3_*x*_4_*x*_5_ = TTCCG and **z** = *z*_1_ *z*_2_*z*_3_*z*_4_*z*_5_ = CCGTT illustrated in Fig. (**3**). We assume that the underlying state sequence is **y** = I_x_I_x_AAAI_z_I_z_ as shown in the figure. As we can see *x*_1_  and *x*_2_ are individually emitted at *I_x_*, hence they are not aligned to any bases in **z** . The pairs (*x*_3_,*z*_1_), (*x*_4_,*z*_2_), and (*x*_5_,*z*_3_) are jointly emitted at *A* , and therefore the bases in the respective pairs are aligned to each other. Finally, *z*_4_ and *z*_5_ are individually emitted at *I_z_* as unaligned bases.

As we can see from this example, there is a one-to-one relationship between the hidden state sequence **y** and the alignment between the two observed sequences **x** and **z** . Therefore, based on the pair-HMM framework, the problem of finding the best alignment between **x** and **z** reduces to the problem of finding the following optimal state sequence

(18) $$y^{*}=a\underset{y}{\mathrm{rgmax}}P\left\{y|x,z,\Theta \right\}.$$

Note that this is identical to finding the optimal path that maximizes *P*{**x,z,y** | Θ}, since we have

(19) $$P\left\{y|x,z,\Theta \right\}=\frac{P\left\{x,z,y|\Theta \right\}}{P\left\{x,z|\Theta \right\}}.$$

The optimal state sequence y^*^ can be found using dynamic programming, by a simple modification of the Viterbi algorithm [3]. The computational complexity of the resulting alignment algorithm is only *O*(*L_x_L_z_*), where *L_x_* and *L_z_* are the lengths of **x** and **z**, respectively.

An important advantage of the pair-HMM based approach over traditional alignment algorithms is that we can use the pair-HMM to compute the alignment probability of a sequence pair. When the given sequences do not display strong similarities, it is difficult to find the correct alignment that is biologically meaningful. In such cases, it would be more useful to compute the probability that the sequences are related, instead of focusing only on their best alignment. The joint observation probability *P*{**x,z** | Θ} of sequences **x** and **z** can be computed by summing over all possible state sequences

(20) $$P\left\{x,z|\Theta \right\}=\sum_{y}P\left\{x,y,z|\Theta \right\}$$

Instead of enumerating all possible state sequences, we can modify the original forward algorithm to compute *P*{**x,z** | Θ} in an efficient manner [3]. It is also possible to compute the alignment probability for individual symbol pairs. For example, the probability that *x_i_* will be aligned to *z_j_* is *P*(*y_k_*= *A* | **x,z,Θ**), where *y_k_* denotes the underlying state for the aligned pair (*x_i_,z_j_*). This probability can be computed as follows

(21) $$P\left\{y_{k}=A|x,z,\Theta \right\}=\frac{P\left\{x_{1}...x_{i},z_{1}...z_{j},y_{k}=A|\Theta \right\}P\left\{x_{i+1}...x_{L_{x},}z_{j+1}...z_{L_{z}}|y_{k}=A,\Theta \right\}}{P\left\{x,z|\Theta \right\}}$$

using a modified forward-backward algorithm [3].

### 4.2. Applications of Pair-HMMs

As pair-HMMs provide a full probabilistic framework for handling pairwise alignments, they have been extensively used for finding pairwise alignment of proteins and DNA sequences [3]. For example, the pair-HMM was used to approximate an explicit model for symbol insertions and deletions (indels) in [48]. The constructed pair-HMM was then used to find the optimal sequence alignment, compute the overall alignment probability, and estimate the reliability of the individual alignment regions. It was demonstrated that using geometrically distributed indel lengths based on pair-HMMs has many potential advantages [48]. More recently, another method called MCALIGN2 [49] also adopted pair-HMMs with a slightly different structure, for global pairwise alignment of noncoding DNA segements. Using pair-HMMs to describe specific indel length distributions has been shown to be very useful for finding accurate alignments of non-coding DNA sequences.

Many multiple sequence alignment (MSA) algorithms also make use of pair-HMMs [50-52]. The most widely adopted strategy for constructing a multiple alignment is the *progressive alignment* approach, where sequences are assembled into one large multiple alignment through consecutive pairwise alignment steps according to a *guide tree* [53, 54]. The algorithms proposed in [50-52] combine pair-HMMs with the progressive alignment approach to construct multiple sequence alignments. For example, the MSA algorithm in [51] uses a pair-HMM to find pairwise alignments and to estimate their alignment reliability. In addition to predicting the best multiple alignment, this method computes the minimum posterior probability for each column, which has been shown to correlate well with the correctness of the prediction. These posterior probabilities can be used to filter out the columns that are unreliably aligned. Another state-of-the-art MSA algorithm called ProbCons [50] also uses a pair-HMM to compute the posterior alignment probabilities. Instead of directly using the optimal alignment predicted by the Viterbi algorithm, ProbCons tries to find the pairwise alignment that maximizes the expected number of correctly aligned pairs based on the posterior probabilities. Furthermore, the algorithm incorporates multiple sequence conservation information when finding the pairwise alignments. This is achieved by using the match quality scores that are obtained from *probabilistic consistency transformation* of the posterior probabilities, when finding the alignments. It was demonstrated that this probabilistic consistency based approach can achieve significant improvement over traditional progressive alignment algorithms [50].

Pair-HMMs have also been used for gene prediction [4, 55-58]. For example, a method called Pairagon+N-SCAN_EST provides a convenient pipeline for gene annotation by combining a pair-HMM with a *de novo* gene prediction algorithm [56]. In this method, a pair-HMM is first used to find accurate alignments of cDNA sequences to a given genome, and these alignments are combined with a gene prediction algorithm for accurate genome annotation. A number of gene-finders adopt a comparative approach for gene prediction [4, 55, 57, 58]. The *generalized pair hidden Markov model (GPHMM)* [4] provides a convenient probabilistic framework for comparative gene prediction by combining the pair-HMM (widely used for sequence alignment and comparison) and the generalized HMM (used by many gene finders). Comparative gene-finders such as SLAM [55] and TWAIN [57] are implemented based on the GPHMM framework. A similar model has been also proposed in [58] to compare two DNA sequences and jointly analyze their gene structures.

Although the pair-HMM is originally defined on the pairwise alignment of *linear* symbol sequences, we can use it for aligning more complex structures, such as trees. For example, the PHMMTSs (pair hidden Markov models on tree structures) extend the pair-HMMs so that we can use them for aligning trees [59]. As most RNA secondary structures can be represented by trees, PHMMTSs provide a useful probabilistic framework for aligning RNA sequences. In [59], PHMMTSs have been used to find the *structural alignment* of RNAs, where an RNA with an unknown structure is aligned to an RNA with a known secondary structure. This structural alignment is distinct from a sequence-based alignment, in the sense that we consider both the structural similarity and the sequence similarity when finding the optimal alignment between the RNAs. *Pair stochastic tree adjoining grammars (PSTAGs)* extend the PHMMTSs further, so that we can use them to align TAG (tree adjoining grammar) trees [60]. This extension allows us to align RNAs with more complicated secondary structures, including pseudoknots.

## 5. CONTEXT-SENSITIVE HMMS AND PROFILE-CSHMMS

Despite their usefulness in various sequence analysis problems, especially, those dealing with proteins and DNA sequences, traditional HMMs have inherent limitations that make them not suitable for handling RNA sequences. Many non-coding RNAs (ncRNAs) conserve base-paired secondary structures that induce pairwise correlations between non-adjacent bases [61]. However, traditional HMMs assume that the emission probability of each symbol depends solely on the underlying state, and since each state depends only on its previous state, they cannot effectively describe correlations between distant symbols. For this reason, more complex models such as the *stochastic context-free grammars (SCFGs)* have been employed in RNA sequence analysis [62, 63]. Although HMMs cannot be directly used for modeling RNAs, we can extend the original model to handle pairwise base correlations. The *context-sensitive HMM (csHMM)* is a variant of HMM that can be used for this purpose [27, 64].

### 5.1. Context-Sensitive Hidden Markov Models

The main difference between a context-sensitive HMM and a traditional HMM is that a csHMM can use part of the past emissions (called the `context') to adjust the probabilities at certain future states. The use of such contextual information is very useful in describing long-range correlations between symbols, and this context-dependency increases the descriptive capability of the HMM considerably [27]. Unlike traditional HMMs, csHMMs use three different types of hidden states: *single-emission states S_n_*, *pairwise-emission states P_n_*, and *context-sensitive states C_n_*. The single-emission states are similar to the regular states in traditional HMMs. They have fixed emission probabilities and do not make use of any contextual information. In addition to the single-emission states, two new types of states, the pairwise-emission states and the context-sensitive states, are introduced in csHMMs. These states cooperate to describe pairwise symbol correlations. Like single-emission states, pairwise-emission states also have fixed emission probabilities. However, the symbols emitted at a pairwise-emission state *P_n_* are stored in the memory ^1^ that is associated with the state *P_n_*. These symbols are used later on as the `contextual information' for adjusting the probabilities at the corresponding context-sensitive state *C_n_*. When we enter the context-sensitive state *C_n_*, we first access the associated memory to retrieve the symbol *x_i_* that was previously emitted at the corresponding pairwise-emission state *y_i_* = *P_n_*. The emission probability at *y_j_* = C_n_(*j* > *i*) is adjusted based on the retrieved symbol *x_i_* (the `context'). We can denote this context-sensitive emission probability as

(22) exj|xi,yi,yj=Pxj is emitted at yj=Cn,given that xi was emitted atyi=Pn

Note that by combining the emission probability *e*(*x_i_*| *y_i_*) at a pairwise-emission state *y_i_* = *P_n_* and the emission probability *e*(*x_j_* | *x_i_*,*y_i_*,*y_j_*) at the corresponding context-sensitive state *y_j_* = *C_n_*, we obtain the joint emission probability of *x_i_* and *x_j_*

(23) $$P\left\{x_{i},x_{j}|y_{i},y_{j}\right\}=P\left\{x_{i}|y_{i}\right\}P\left\{x_{j}|x_{i},y_{i},y_{j}\right\}=e\left(x_{i}|y_{j}\right)e\left(x_{j}|x_{i},y_{i},y_{j}\right),$$

where we used the fact that *x_i_* is independent of *y_i_*. This clearly shows that we can describe long-range pairwise symbol correlations by using a pair of *P_n_* and *C_n_*, and then specifying their emission probabilities. Since a given pairwise-emission state *C_n_* and its corresponding context-sensitive state *C_n_* work together to describe the symbol correlations, these states always exist in pairs, and a separate memory is allocated to each state pair (*P_n_*, *C_n_*). As we need the contextual information to adjust the emission probabilities at a context-sensitive state, the transition probabilities in the model are adjusted such that we never enter a context-sensitive state if the associated memory is empty [27].

Using context-sensitive HMMs, we can easily describe any kind pairwise symbol correlations by arranging the pairwise emission states *P_n_* and the corresponding context-sensitive states *C_n_* accordingly. As a simple example, let us consider a csHMM that generates *only* symmetric sequences, or palindromes. Such an example is shown in Fig. (**4**).

The model has three states, a pair of pairwise-emission state *P_1_* and context-sensitive state *C_1_*, and one single-emission state *S_1_*. In this example, the state pair (*P_1_*, *C_1_*) uses a stack, and the two states work together to model the symbol correlations that are induced by the symmetry of the sequence. Initially, the csHMM enters the pairwise-emission state *P_1_* and emits one or more symbols. The symbols emitted at *P_1_* are stored in the stack. When we enter *C_1_*, we first retrieve a symbol from the top of the stack. Based on this symbol, the emission probabilities of *C_1_* are adjusted such that it emits an identical symbol with probability 1. Transition probabilities of *C_1_* are adjusted such that it makes a transition to itself until the stack becomes empty. Once the stack becomes empty, the csHMM terminates. In this way, the csHMM shown in Fig. (**4**) generates only palindromes that take one of the following forms

x*_e_* = *x*_1_*x*_2_ ... *x_N_x_N_* ... *x*_2_*x*_1_ (*even length*)

x*_o_* = *x*_1_*x*_2_ ... *x_N_x_N+1_x_N_* ... *x*_2_*x*_1_ (*odd length*)

The underlying state sequences for **x**_*e*_ and **x**_*o*_ will be

ye=P1...P1︸N statesC1...C1︸N states and yo=P1...P1︸S1N statesC1...C1︸,N states

respectively. Note that the single-emission state *S_1_* is only used to generate the symbol located in the center of a palindrome with odd length, since this symbol is not correlated to any other symbols.

This example clearly shows how we can represent pairwise correlations using a csHMM. When modeling RNAs with conserved base-pairs, we can arrange *P_n_* and *C_n_* based on the positions of the base-pairs, and adjust the emission probabilities at *C_n_* such that they emit the bases that are complementary to the bases emitted at the corresponding *P_n_*. By adjusting the context-sensitive emission probabilities *e*(*x_j_* | *x_i_*, *y_i_* = *P_n_*, *y_j_* = *C_n_*), we can model any kind of base-pairs including non-canonical pairs. Considering that the widely used stochastic context-free grammars can model only nested base-pairs, hence no pseudoknots, the increased modeling capability and the ease of representing any kind of base-paired structures are important advantages of context-sensitive HMMs [9, 61].

### 5.2. Profile Context-Sensitive HMMs

Suppose we have a multiple alignment of relevant RNA sequences. How can we build a probabilistic model to represent the RNA profile, or the important features in the given RNA alignment? Due to the conservation of secondary structure, multiple RNA alignments often display column-wise correlations. When modeling an RNA profile, it is important to reflect these correlations in the model, along with the conserved sequence information. The *profile context-sensitive HMM (profile-csHMM)* provides a convenient probabilistic framework that can be used for this purpose [9, 65]. Profile-csHMMs are a subclass of context-sensitive HMMs, whose structure is similar to that of profile-HMMs. As it is relatively simple to construct a profile-HMM from a protein or DNA sequence alignment, it is rather straightforward to build a profile-csHMM based on a multiple RNA alignment with structural annotation.

Like conventional profile-HMMs, profile-csHMMs also repetitively use *match states M_k_*, *insert states I_k_*, and *delete states D_k_* to model symbol matches, symbol insertions, and symbol deletions, respectively. The main difference between a profile-HMM and a profile-csHMM is that the profile-csHMM can have three different types of match states. As we have seen in Sec. 5.1, context-sensitive HMMs use three different types of states, where the single-emission states *S_n_* are used to represent the symbols that are not directly correlated to other symbols, while the pairwise-emission states *P_n_* and the context-sensitive states *C_n_* are used together to describe pairwise symbol correlations. In a profile-csHMM, each *M_k_* can choose from these three types of states. Therefore, we can have *single-emission match states*, *pairwise-emission match states*, and *context-sensitive match states*. Single-emission match states are used to represent the columns that are not involved in base-pairing. The pairwise correlations between columns, induced by conserved base-pairs, can be represented by using pairwise-emission match states and the corresponding context-sensitive match states.

As an example, let us assume that we want to construct a profile-csHMM for the alignment shown in Fig. (**5a**). Since the alignment has five columns, we need five match states to represent the sequence profile. There exist two base-pairs in the consensus RNA structure, where the bases in the first column form base-pairs with those in the fourth column, and the bases in the second column form base-pairs with those in the fifth column. In order to describe the correlation between the first and the fourth columns, we use a pairwise-emission state for the first match state *M_1_* and the corresponding context-sensitive state for the fourth match state *M_4_*. Similarly, we use a pairwise-emission state for *M_2_* and the corresponding context-sensitive state for *M_5_*. We use a single-emission state for the third match state *M_3_*, since the third column is not involved in base-pairing. By interconnecting the five match states *M_1_*,*M_2_*,...,*M_5_*, we obtain an *ungapped* csHMM for the given alignment, as shown in Fig. (**5b**). Finally, we add insert states *I_k_* and delete states *D_k_* to the ungapped model to obtain the final profile-csHMM. Since the inserted bases are not correlated to other bases, we use a single-emission state for each *I_k_*. As in profile-HMMs, the delete states *D_k_* are non-emitting states, and they are simply used to interconnect the neighboring states.

As illustrated in this example, profile-csHMMs provide a convenient tool of modeling RNA profiles. Profile-csHMMs can represent *any* kind of base-pairs by appropriately arranging the pairwise-emission match states and the context-sensitive match states. Due to the increased descriptive capability, algorithms for traditional HMMs (e.g., the Viterbi algorithm) cannot be directly used for profile-csHMMs. However, we can generalize these algorithms so that they can be used with profile-csHMMs. For example, the *sequential component adjoining (SCA)* algorithm [9], which is a generalization of the Viterbi algorithm, provides a systematic way of finding the optimal state sequence in a profile-csHMM.

### 5.3. Hidden Markov Models in RNA Sequence Analysis

Profile-csHMMs can be used for finding structural alignment of RNAs and performing RNA similarity searches [9, 66]. In [9], the profile-csHMM has been used to find the optimal alignment between a folded RNA (and RNA with a known secondary structure) and an unfolded RNA (an RNA whose folding structure is not known). To find the structural alignment between the two RNAs, we first construct a profile-csHMM to represent the folded RNA. The parameters of the profile-csHMM is chosen according to the scoring scheme proposed in [67]. Based on this model, we use the SCA algorithm to find the optimal state sequence that maximizes the observation probability of the unfolded RNA sequence. The optimal alignment between the two RNAs can be unambiguously determined from the predicted state sequence. Furthermore, we can infer the secondary structure of the unfolded RNA based on the alignment. Theoretically, the profile-csHMM based RNA structural alignment method can handle any kind of pseudoknots. The current implementation of the algorithm [9] can align any RNAs in the Rivas&Eddy class [68] that includes most of the known RNAs [69]. We may use this structural alignment approach for building RNA similarity search tools.

One practical problem that frequently arises in RNA sequence analysis is the high computational complexity. As RNA alignment algorithms have to deal with complicated base-pair correlations, they require significantly more computations compared to sequence-based alignment algorithms. For example, the *Cocke-Younger-Kasami (CYK) algorithm* [3], which is the SCFG analogue of the Viterbi algorithm for HMMs, has a complexity of *O*(*L^3^*), where *L* is the length of the RNA to be aligned. Considering that the computational complexity of the Viterbi algorithm increases only linearly with the sequence length, this is a significant increase. The complexity of a simultaneous RNA folding (structure prediction) and alignment algorithm [70] is even higher, and they need *O*(*L^3N^*) computations for aligning *N* RNAs of length *L* . These algorithms do not consider pseudoknots, and if we allow pseudoknots, the complexity will increase further. The high computational cost often limits the utility of many RNA sequence analysis algorithms, especially when the RNA of interest is long.

To overcome this problem, various heuristics have been developed to expedite RNA alignment and RNA search algorithms. For example, profile-HMM based prescreening filters [11, 71] have been proposed to improve the speed of RNA searches based on *covariance models (CMs)*. Covariance models can be viewed as profile-SCFGs that have a special structure useful for modeling RNA families [3, 63]. In this prescreening approach [11, 71], we first construct a profile-HMM based on the CM that is to be used in the homology search. Note that the resulting profile-HMM conveys only the consensus sequence information of the RNA family represented by the given CM. This profile-HMM is then used to prescreen the genome database to filter out the sequences that are not likely to be annotated as homologues by this CM. The complex CM is run only on the remaining sequences, thereby reducing the average computational cost. It has been demonstrated that using profile-HMM prescreening filters can make the search hundreds of times faster at no (or only a slight) loss of accuracy. A similar approach can be used to speed up profile-csHMM based RNA searches [72].

There also exist a number of methods to improve the speed of simultaneous RNA folding and alignment algorithms [10, 73]. For example, Consan implements a constrained version of the pairwise RNA structure prediction and alignment algorithm based on *pair stochastic context-free grammars (pair-SCFGs)* [73]. It assumes the knowledge of a few confidently aligned base position, called `pins', which are fixed during the alignment process to reduce the overall complexity. These pins are chosen based on the posterior alignment probabilities that are computed using a pair-HMM. A recent version of another pairwise folding and alignment algorithm called Dynalign [10] also employs alignment constraints to improve its efficiency. Dynalign also uses a pair-HMM to compute the posterior alignment and insertion probabilities, which are added to obtain the so-called co-incidence probabilities. We estimate the set of alignable base positions by thresholding the co-incidence probabilities, and this set is subsequently used to constrain the pairwise RNA alignment. It has been shown that employing these alignment constraints can significantly reduce the computational and memory requirements without degrading the structure prediction accuracy [10, 73].

## 6. CONCLUDING REMARKS

Hidden Markov models have become one of the most widely used tools in biological sequence analysis. In this paper, we reviewed several different types of HMMs and their applications in molecular biology. It has to be noted that this review is by no means exhaustive, and that there still exist many other types of HMMs and an even larger number of sequence analysis problems that have benefited from HMMs. Hidden Markov models provide a sound mathematical framework for modeling and analyzing biological sequences, and we expect that their importance in molecular biology as well as the range of their applications will grow only further.

## Figures and Tables

**Fig. (1):**
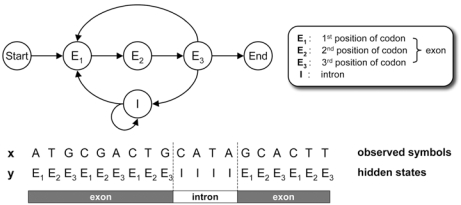
A simple HMM for modeling eukaryotic genes.

**Fig. (2):**
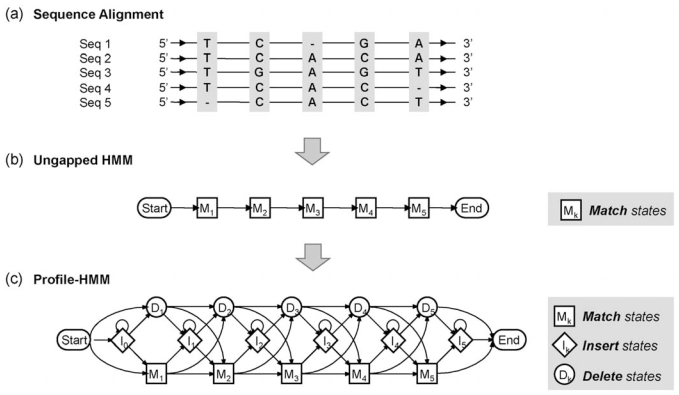
Profile hidden Markov model. (**a**) Multiple sequence alignment for constructing the profile-HMM. (**b**) The ungapped HMM that represents the consensus sequence of the alignment. (**c**) The final profile-HMM that allows insertions and deletions.

**Fig. (3):**
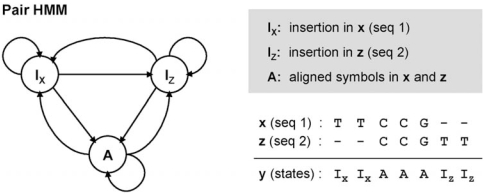
Example of a pair hidden Markov model. A pair-HMM generates an aligned pair of sequences. In this example, two DNA sequences **x** and **z** are simultaneously generated by the pair-HMM, where the underlying state sequence is **y**. Note that the state sequence **y** uniquely determines the pairwise alignment between **x** and **z** .

**Fig. (4):**
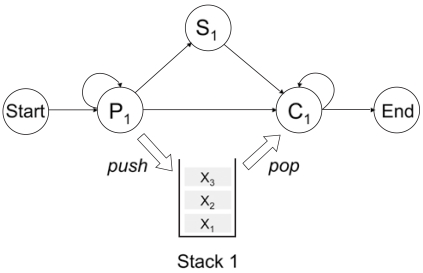
A context-sensitive HMM that generates only symmetric sequences, or palindromes.

**Fig. (5):**
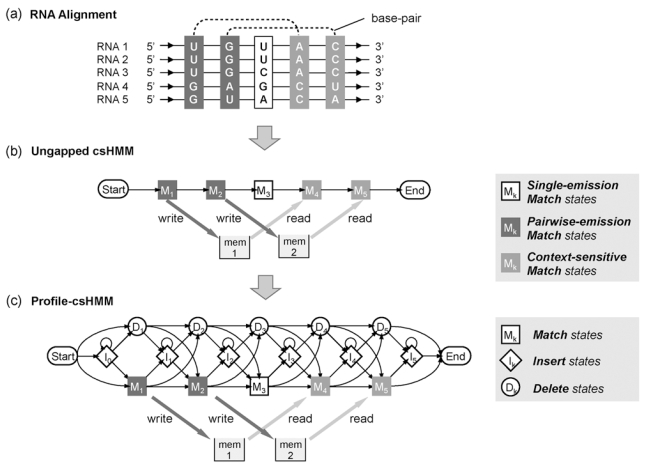
Constructing a profile-csHMM from a multiple RNA sequence alignment. (**a**) Example of an RNA sequence alignment. The consensus RNA structure has two base-pairs. (**b**) An ungapped csHMM constructed from the given alignment. (**c**) The final profile-csHMM that can handle symbol matches, insertions, and deletions.

## Footnotes

1 Although different types of memories (stacks, queues, etc.) can be used with csHMMs, it is convenient to use stacks for modeling RNAs.
